# Obesity and Oral Health‐Related Quality of Life: An Exploratory Analysis of Associated Factors

**DOI:** 10.1111/odi.70326

**Published:** 2026-04-10

**Authors:** Ana Karina de Oliveira de Santana Barros, Isaac Suzart Gomes‐Filho, Simone Seixas da Cruz, Soraya Castro Trindade, Samilly Silva Miranda, Ana Claudia Morais Godoy Figueiredo, Daline Oliveira Carneiro, Maria Clara Azevedo Moreira dos Santos, Isabela de Souza Sacramento, Eneida de Moraes Marcílio Cerqueira, Josicélia Estrela Tuy Batista, Alexandre Marcelo Hintz, Gregory John Seymour, Frank Andrew Scannapieco, Peter Michael Loomer, Johelle de Santana Passos‐Soares

**Affiliations:** ^1^ Department of Health Feira de Santana State University Feira de Santana Bahia Brazil; ^2^ Health Sciences Center Federal University of Recôncavo of Bahia Santo Antônio de Jesus Bahia Brazil; ^3^ Collective Health Institute Federal University of Bahia Salvador Bahia Brazil; ^4^ Epidemiology Surveillance Federal District Health State Department Brasília Brazil; ^5^ Department of Preventive Dentistry, Faculty of Dentistry Federal University of Bahia Salvador Brazil; ^6^ School of Dentistry The University of Queensland Herston Queensland Australia; ^7^ Department of Oral Biology University at Buffalo Buffalo New York USA; ^8^ School of Dentistry University of Texas Health Science Center at San Antonio San Antonio Texas USA

**Keywords:** associated factors, epidemiology, obesity, quality of life, tooth loss

## Abstract

**Objectives:**

This study assessed oral health‐related quality of life in individuals with and without obesity, and investigated its association with socioeconomic‐demographic characteristics, lifestyle habits, health conditions, and clinical care.

**Methods:**

A cross‐sectional study was conducted with 250 individuals (≥ 18 years) in Salvador‐Brazil. Data were collected using structured questionnaires, anthropometric assessments, and clinical oral examinations. Oral health‐related quality of life was measured using the Oral Health Impact Profile‐14. Obesity was classified by waist circumference. Sequential blockwise multiple regression models were employed for data analysis.

**Results:**

Impaired oral health‐related quality of life was reported by 56.7% of the overall sample. Obese individuals showed worse Oral Health Impact Profile‐14 scores compared to non‐obese individuals, with greater impairment in physical pain, and physical, psychological, and social disability domains. In final multivariate models, female sex, cardiovascular disease, and the decayed, missing, and filled teeth index were associated with impaired oral health‐related quality of life among obese individuals.

**Conclusions:**

Obese individuals showed lower oral health‐related quality of life scores than non‐obese individuals, linked to poor oral health. Female sex, cardiovascular disease, and high decayed, missing, and filled teeth index scores emerged as significant factors associated with worse outcomes.

## 1 Introduction

Obesity is a chronic, multifactorial inflammatory disease characterized by excessive body fat accumulation due to an imbalance between calorie intake and expenditure (Mayoral et al. 2020). It represents a growing global public health problem and is associated with multiple comorbidities, including diabetes, depression, cancer, cardiovascular conditions such as hypertension and myocardial infarction, and oral diseases (Felisbino‐Mendes et al. 2020; GBD 2017 Disease and Injury Incidence and Prevalence Collaborators 2018).

The most widely used and practical criterion for diagnosing obesity is body mass index (BMI ≥ 30 kg/m^2^) (WHO 1995). However, BMI does not account for fat distribution and may be influenced by sex, age, and ethnicity (Buss 2014). As visceral fat is particularly linked to increased health risks, additional measurements such as abdominal circumference (AC) are recommended to complement diagnosis (Alberti et al. 2006; National Cholesterol Education Program (NCEP) Expert Panel on Detection, Evaluation, and Treatment of High Blood Cholesterol in Adults (Adult Treatment Panel III) 2002).

Obesity results from a combination of genetic, behavioral, and environmental factors, including poor diet, physical inactivity, stress, and exposure to endocrine disruptors (Mayoral et al. 2020; Reddon et al. 2016). It can negatively affect quality of life, especially when combined with socioeconomic factors such as low income, educational level, and occupation (Wang et al. 2022; Kolotkin and Andersen 2017).

Despite well‐established links between obesity and general health, few studies have evaluated the oral health‐related quality of life (OHRQoL) in obese individuals (Khongsirisombat et al. 2022; do Prado et al. 2020), or examined associated factors. Most OHRQoL research focuses on other chronic diseases such as cardiovascular conditions, diabetes, and kidney disease (Lazureanu et al. 2022; Ruokonen et al. 2019). Evidence indicates that individuals with obesity tend to have poorer oral health, with higher rates of caries, tooth loss, and periodontal disease, and experience greater impairment in their quality of life compared to non‐obese individuals (Khongsirisombat et al. 2022; Arslan 2023; Carneiro et al. 2022).

Impaired oral function, including pain and discomfort during eating, may compromise nutritional intake and lead to deficiencies that impair immunity and tissue healing (Almoznino et al. 2020).

OHRQoL is a multidimensional construct reflecting individuals' perceptions of how oral health impacts their daily lives (Petersen and Ogawa 2018). The Oral Health Impact Profile‐14 (OHIP‐14) is a widely used instrument to assess this, which evaluates seven dimensions of psychological and behavioral impact from oral conditions (Locker et al. 2001; Slade 1997). Originally developed with 49 items, it was later shortened to 14 while maintaining reliability (Locker and Gibson 2006).

Understanding patients' perceptions about their oral health and its influence on quality of life provides insights that go well beyond clinical data. These perspectives can help to inform better clinical strategies, planning of health services, and public policies.

Given the scarcity of research on OHRQoL in obese populations, this study aimed to assess OHRQoL in individuals with and without obesity and to investigate its associations with demographic, socioeconomic, lifestyle, and health‐related factors. We hypothesize that individuals with obesity experience worse OHRQoL, influenced by socioeconomic status, health conditions, and lifestyle habits.

## 2 Materials and Methods

### 2.1 Study Design and Sample

This cross‐sectional study was conducted with individuals aged 18 years or older, attending public health services in Salvador, Brazil, between 2016 and 2019. The research was approved by the Research Ethics Committee of the State University of Feira de Santana (CAEE 42744415.0.0000.0053) and was conducted in accordance with the Declaration of Helsinki of 1975, revised in 2013.

Individuals with the following conditions were excluded from the study: (1) received periodontal treatment within the 6 months before the study; (2) neoplasms; (3) immune compromised and/or HIV/AIDS; (4) pregnant women; and (5) unable to comprehend or respond to the questionnaire.

For sample size calculation, the following parameters were assumed: 5% margin of error, 80% power, and mean (standard deviation) OHIP‐14 scores of 12.55 (±7.35) for individuals with moderate periodontitis and 9.53 (±7.12) for individuals without periodontitis (Al Habashneh et al. 2012). The calculated minimum required sample size to detect significant differences in quality of life was 182 individuals.

Upon obtaining written informed consent, all participants completed questionnaires to gather data on sociodemographic characteristics, lifestyle habits, health conditions, and oral health care practices, as well as the OHIP‐14 instrument.

### 2.2 Physical and Oral Examination

Physical examinations were conducted in a standardized manner to obtain anthropometric measurements (waist circumference [WC], weight, and height). WC was measured using a non‐elastic tape measure (150 cm length, 0.5 cm scale, TBW brand), with the individual standing upright, maintaining a straight posture, relaxed abdominal muscles, and breathing normally. The tape measure was placed at the distance between the iliac crest and the lower rib margin (WHO 2017).

Height was measured using a portable stadiometer, fixed to a flat surface, with an accuracy of 0.1 cm. Participants were instructed to stand upright, with their feet parallel and shoulder‐width apart, and arms relaxed at their sides.

Weight was measured using a digital scale (Techline brand) with a capacity of 180 kg and an accuracy of 100 g. The scale was placed on a flat, hard, and level surface, and participants were positioned in the center of the scale, wearing light clothing and barefoot.

Oral examinations were performed by a trained dentist to assess the following measurements: probing depth (Pihlstrom et al. 1981), recession or hypertrophy index, clinical attachment loss (Ramfjord 1959), bleeding on probing (Ainamo and Bay 1976), plaque index (Ainamo and Bay 1976), decayed, missing, and filled teeth index (DMFT), and the number of remaining teeth. The periodontal examination was performed at six sites of each existing tooth, excluding third molars (Pihlstrom et al. 1981; Löe 1967).

Periodontitis diagnosis was determined based on criteria from the Centers for Disease Control and Prevention (CDC) and the American Academy of Periodontology (AAP) (Eke et al. 2012). Participants were classified as having periodontitis if they met one of three severity conditions: mild, moderate, or severe.

The DMFT index, recommended by the World Health Organization (WHO) to measure and compare dental caries experience in population groups, was used to assess dental caries in permanent teeth. The index expresses the mean number of decayed, missing, and filled teeth. The criteria and codes for the DMFT index were established by the SB Brazil Project (Brasil. Ministério da Saúde. Secretaria de Atenção à Saúde. Departamento de Atenção Básica 2006) and WHO guidelines for epidemiological surveys (Petersen and Baez 2013). Dental caries classification in this study considered both the occurrence and severity of the condition. Caries occurrence was defined by the number of teeth with caries lesions or restored teeth with caries. Caries severity was based on the distribution of the DMFT. These two classifications for dental caries used quartile‐based cut‐offs (WHO 2017). Regarding remaining teeth, participants were categorized according to the terciles of the data distribution for remaining teeth.

### 2.3 Obesity Diagnosis

Obesity was defined according to the cut‐off points established by two anthropometric criteria. Criterion 1 adopted the obesity thresholds established by the International Diabetes Federation (IDF): WC ≥ 90 cm for men and ≥ 80 cm for women (recommended thresholds for South American populations) (Alberti et al. 2009). Individuals below these cut‐off points were categorized as non‐obese.

Criterion 2 followed the waist circumference cut‐off points recommended by the National Cholesterol Education Program (NCEP‐ATPIII), which defines abdominal obesity as WC ≥ 102 cm for men and ≥ 88 cm for women (National Cholesterol Education Program (NCEP) Expert Panel on Detection, Evaluation, and Treatment of High Blood Cholesterol in Adults (Adult Treatment Panel III) 2002). The use of both IDF and NCEP‐ATPIII criteria followed the original research protocol approved by the Institutional Review Board (IRB) and the requirements of the funding agencies. This dual assessment was designed to compare the associations of OHRQoL with different validated thresholds of central adiposity, considering that IDF criteria are more sensitive (lower thresholds), while NCEP‐ATPIII criteria are more specific to metabolic risk in Western populations.

### 2.4 Outcome Variable—Oral Health‐Related Quality of Life (OHRQoL)

OHRQoL was assessed using the Oral Health Impact Profile‐14 (OHIP‐14) instrument, which consists of 14 items distributed across 7 domains: functional limitations, physical incapacity, psychological incapacity, physical pain, psychological discomfort, social incapacity, and physical limitation. The score for each item was calculated using a 5‐point scale (0 for never to 4 for always). The total OHIP‐14 score ranges from 0 to 56 (Slade 1997). A higher score indicates a greater impairment of OHRQoL.

These scores were transformed into prevalence by considering the percentage of individuals who responded with “constantly” or “always” to one or more items (Slade et al. 2005). The response patterns were categorized into impaired OHRQoL (constantly/always) and non‐impaired OHRQoL (never/rarely/sometimes).

This variable was also assessed according to its median cutoff.

### 2.5 Independent Variables

The following variables were analyzed as predictors of OHRQoL among obese and non‐obese individuals: sex (female, male), age in years (categorized as 18–52 years; > 52 years), education level (in years of schooling: > 4 years, ≤ 4 years), race/skin color (white; non‐white), family income (less than 1 minimum wage; ≥ 1 minimum wage); occupation (employed, unemployed); self‐reported diagnosis of diabetes (yes, no); cardiovascular disease (CVD) (yes, no); hypertension (yes, no); smoking habit (current smoker/former smoker; non‐smoker); alcoholic beverage consumption (yes; no); physical activity practice (yes; no); time since the last dental visit (< 1 year; ≥ 1 year); frequency of tooth brushing per day (> 2 times; ≤ 2 times); daily dental floss use (yes; no); clinical attachment level of 3 to 4 mm (< 30% of teeth; ≥ 30% of teeth); clinical attachment level ≥ 5 mm (< 30% of teeth; ≥ 30% of teeth); plaque index (< 38%; ≥ 38%); probing depth (teeth with at least one site < 4 mm; ≥ 4 mm); gingival bleeding (< 10%; ≥ 10%); remaining teeth (> 11 teeth; ≤ 11 teeth); presence of caries (< 2 decayed teeth; ≥ 2 decayed teeth); caries severity (CPOD < 16; CPOD ≥ 16).

### 2.6 Data Analysis Procedures

Initially, descriptive analysis was conducted to examine the general distribution of the frequency of the impact on quality of life and mean OHIP‐14 scores. Categorical variables were described by their frequency distributions, while numerical variables were described using measurements of central tendency and dispersion (mean, standard deviation, median, and interquartile range).

For the bivariate analysis between independent variables and OHRQoL, the Chi‐square test or Fisher's Exact test was employed. For continuous variables, the Mann–Whitney test was used after confirming non‐normality with the Kolmogorov–Smirnov test. A significance level of 5% was used.

Associations between independent variables and OHRQoL were analyzed, stratified by the three obesity criteria, generating three final multiple regression models.

To identify factors associated with OHRQoL, a sequential blockwise multiple regression model was used. The distal level consisted of the block of sociodemographic characteristics (Block I); the intermediate level (Block II) included lifestyle habits and health care practices; and the proximal level included general health conditions and clinical oral health (Block III). Logistic regression was applied to calculate association measurements, and subsequently, odds ratios were converted into prevalence ratios (PR) with their 95% confidence intervals (95% CI) through Poisson regression with robust variance.

The selection of independent variables for inclusion in the multiple regression model was based on bivariate analyses of each block. Variables with a *p*‐value < 0.20 were selected for multiple analysis. The distal block was the first to enter the model, serving as a control factor for the subsequent blocks. The remaining blocks were then added. Variables with a *p*‐value ≤ 0.05 were considered statistically significant. For each model, the remaining independent/predictor variables were included as adjustment variables.

A quartile regression analysis was employed to analyze the OHRQoL outcome, considering the median as the cutoff point.

Multicollinearity diagnosis was performed using the variance inflation factor (VIF). The model was assessed with the deviance test, where a *p*‐value > 0.05 indicated a good model fit. Analyses were performed using SPSS and STATA^1^ statistical software.

## 3 Results

The sample was comprised of 254 individuals, with a mean age of 50 years (±13.71 standard deviation [SD]), consisting of 70 men and 184 women. The prevalence of obesity varied depending on the criterion used: 52.4% (*n* = 133, as defined by the NCEP criterion) and 79.9% (*n* = 203, as defined by the IDF criterion). Regarding oral health conditions, it was observed that 39.4% of the overall sample had at least two decayed teeth (highest quartile), and 74.8% had periodontitis, with moderate periodontitis being the most prevalent (50%). No statistically significant differences were found in oral health conditions between the obesity criteria adopted (data not shown).

The overall mean score of OHIP‐14 and the prevalence of impaired OHRQoL, as defined by the affirmative responses “almost always” or “very frequently,” were 15.2 (±11.9 SD) and 56.7%, respectively. The overall median OHIP‐14 score was 12.0 (lower quartile 6; upper quartile 23). Table 1 presents the means (±SD), medians (interquartile range [IQR]), and prevalences for each item of the OHIP‐14 and of the impaired OHRQoL in the overall sample. The highest prevalences were observed in the domains “psychological discomfort” (concern about oral problems—35.8%), “psychological disability” (embarrassment—27.6%), and “physical pain” (discomfort when eating—25.6%).

**TABLE 1:** Distribution of OHIP‐14 scores and Prevalence of Impaired Oral Health‐Related Quality of Life (OHRQoL) in the Overall Sample.

Dimensions and Items	Impaired OHRQoL *n* (%)	Mean (±SD)	Median (IQR)
Total OHIP‐14	144 (56.7)	15.2 (12.0)	12.0 (6.0–23.0)
Functional Limitation			
Problems in Pronouncing Words	33 (13.0)	1.0 (1.3)	0.0 (0.0–2.0)
Change in Taste	23 (9.1)	0.8 (1.2)	0.0 (0.0–2.0)
Physical Pain			
Severe Pain when Eating	45 (17.7)	1.5 (1.2)	2.0 (0.0–2.0)
Discomfort when Eating	65 (25.6)	1.5 (1.3)	2.0 (0.0–3.0)
Psychological Discomfort			
Worry about Oral Problems	91 (35.8)	1.9 (1.5)	2.0 (0.0–3.0)
Stress from Oral Problems	45 (17.7)	1.2 (1.3)	1.0 (0.0–2.0)
Physical Inability			
Unsatisfactory Diet	59 (23.2)	1.3 (1.4)	1.0 (0.0–2.0)
Interrupted Meals	31 (12.2)	0.9 (1.2)	0.0 (0.0–2.0)
Psychological Disability			
Difficulty Relaxing	32 (12.6)	0.9 (1.2)	0.0 (0.0–2.0)
Feeling of Embarrassment	70 (27.6)	1.5 (1.5)	1.0 (0.0–3.0)
Social Inability			
Irritability	27 (10.6)	0.7 (1.2)	0.0 (0.0–1.0)
Difficulty Performing Daily Activities	17 (6.7)	0.6 (1.1)	0.0 (0.0–1.0)
Social Disadvantage			
Perception of Less Satisfactory Life	35 (13.8)	0.8 (1.3)	0.0 (0.0–2.0)
Unable to Perform Activities	13 (5.1)	0.5 (0.9)	0.0 (0.0–0.0)

Abbreviations: IQR, Interquartile Range; SD, Standard Deviation.

The number of individuals classified as obese (NCEP criterion) was more numerous (63.2%) than those classified as non‐obese (49.6%) (*p* = 0.03). The OHIP‐14 items that showed statistically significant associations with obesity according to this criterion were: discomfort while eating (*p* = 0.02), unsatisfactory diet (*p* = 0.03), feelings of embarrassment (*p* = 0.01), irritability (*p* = 0.02), and difficulty performing daily activities (*p* = 0.04) (Table 2). No associations were observed for the IDF criterion, although the highest mean was observed among obese individuals.

**TABLE 2:** Bivariate Comparison of Frequencies and Mean OHIP‐14 Scores by Item and Domain Between Obese and Non‐Obese Groups, Stratified by IDF and NCEP Criteria.

OHIP‐14 domains	Non‐Obese IDF	Obese IDF	*p* *	Non‐Obese NCEP	Obese NCEP	*p* *
	(*n* = 51)	(*n* = 203)		(*n* = 121)	(*n* = 133)	
General (*n*, %)	26 (51.0)	118 (58.1)	0.36	60 (49.6)	84 (63.2)	**0.03**
Mean (±SD)	14.1 (11.9)	15.5 (12.0)	0.36	13.8 (11.6)	16.5 (12.2)	**0.04**
Functional Limitation						
Problems in Pronouncing Words (*n*, %)	7 (13.7)	26 (12.8)	0.86	13 (10.7)	20 (15.0)	0.31
Mean (±SD)	0.8 (1.3)	1.0 (1.3)	0.43	0.8 (1.3)	1.1 (1.3)	0.23
Change in Taste (*n*, %)	2 (3.9)	21 (10.3)	0.15	11 (9.1)	12 (9.0)	0.99
Mean (±SD)	0.7 (1.0)	0.8 (1.2)	0.90	0.8 (1.2)	0.7 (1.2)	0.43
Physical Pain						
Severe Pain when Eating (*n*, %)	9 (17.6)	36 (17.7)	0.99	18 (14.9)	27 (20.3)	0.26
Mean (±SD)	1.5 (1.2)	1.6 (1.3)	0.73	1.4 (1.2)	1.7 (1.2)	0.09
Discomfort when Eating (*n*, %)	14 (27.5)	51 (25.1)	0.73	27 (22.3)	38 (28.6)	0.25
Mean (±SD)	1.5 (1.4)	1.6 (1.3)	0.60	1.4 (1.3)	1.8 (1.3)	**0.02**
Psychological Discomfort						
Worry about Oral Problems (*n*, %)	18 (35.3)	73 (36.0)	0.93	37 (30.6)	54 (40.6)	0.10
Mean (±SD)	1.8 (1.6)	1.9 (1.4)	0.63	1.8 (1.5)	2.0 (1.4)	0.16
Stress from Oral Problems (*n*, %)	11 (21.6)	34 (16.7)	0.42	20 (16.5)	25 (18.8)	0.64
Mean (±SD)	1.3 (1.5)	1.2 (1.3)	0.90	1.2 (1.3)	1.3 (1.4)	0.56
Physical Inability						
Unsatisfactory Diet (*n*, %)	7 (13.7)	52 (25.6)	0.07	21 (17.4)	38 (28.6)	**0.03**
Mean (±SD)	1.0 (1.3)	1.3 (1.4)	0.10	1.1 (1.3)	1.4 (1.5)	0.19
Interrupted Meals (*n*, %)	6 (11.8)	25 (12.3)	0.91	13 (10.7)	18 (13.5)	0.5
Mean (±SD)	0.8 (1.1)	0.9 (1.2)	0.36	0.8 (1.1)	0.9 (1.2)	0.36
Psychological Disability						
Difficulty Relaxing (*n*, %)	7 (13.7)	25 (12.3)	0.79	15 (12.4)	17 (12.8)	0.93
Mean (±SD)	1.0 (1.2)	0.9 (1.2)	0.24	0.9 (1.2)	0.9 (1.3)	0.71
Feeling of Embarrassment (*n*, %)	14 (27.5)	56 (27.6)	0.99	24 (19.8)	46 (34.6)	**0.01**
Mean (±SD)	1.4 (1.6)	1.5 (1.5)	0.65	1.2 (1.4)	1.7 (1.6)	**0.01**
Social Inability						
Irritability (*n*, %)	4 (7.8)	23 (11.3)	0.46	7 (5.8)	20 (15.0)	**0.02**
Mean (±SD)	0.6 (1.1)	0.8 (1.2)	0.34	0.6 (1.0)	0.9 (1.4)	0.12
Difficulty Performing Daily Activities (*n*, %)	4 (7.8)	13 (6.4)	0.71	4 (3.3)	13 (9.8)	**0.04**
Mean (±SD)	0.6 (1.1)	0.8 (1.2)	0.70	0.5 (0.9)	0.7 (1.1)	0.44
Social Disadvantage						
Perception of Less Satisfactory Life (*n*, %)	5 (9.8)	30 (14.8)	0.36	17 (14.0)	18 (13.5)	0.91
Mean (±SD)	0.7 (1.2)	0.8 (1.3)	0.66	0.8 (1.2)	0.9 (1.3)	0.28
Unable to Perform Activities (*n*, %)	2 (3.9)	11 (5.4)	0.66	4 (3.3)	9 (6.8)	0.21
Mean (±SD)	0.3 (0.8)	0.5 (0.9)	0.06	0.3 (0.8)	0.5 (1.0)	0.21

*Note:* Bold numbers indicate a statistically significant difference.

Abbreviations: IDF, International Diabetes Federation; NCEP, National Cholesterol Education Program; SD, standard deviation.

* *p* value = Statistical significance level: *p* ≤ 0.05.

When the obesity criterion according to the IDF was applied (Table 3), obese individuals, females, those with cardiovascular disease, and those with lower tooth brushing frequency had higher percentages of impaired OHRQoL (*p* < 0.05). Additionally, obese individuals with higher plaque index (*p* = 0.01), fewer remaining teeth (*p* = 0.05), and a DMFT ≥ 16 (*p* = 0.01) also showed worse OHRQoL (Table S1).

**TABLE 3:** Bivariate Association of Oral Health‐Related Quality of Life (OHRQoL) with Socio‐demographic, Lifestyle, and Health Conditions, Stratified by Obesity Status (IDF and NCEP Criteria).

	Non‐obese – IDF (*n* = 51)		Obese – IDF (*n* = 203)		Non‐obese – NCEP (*n* = 121)		Obese – NCEP (*n* = 133)	
	Compromised OHRQoL	Mean (±SD)	Compromised OHRQoL	Mean (±SD)	Compromised OHRQoL	Mean (±SD)	Compromised OHRQoL	Mean (±SD)
Sex								
Male	13 (54.2)	12.1 (9.8)	19 (41.3)	12.5 (10.6)	20 (39.2)	11.5 (10.1)	12 (63.2)	14.6 (10.7)
Female	13 (48.1)	15.8 (13.5)	99 (63.1)	16.4 (13.0)	40 (57.1)	15.4 (12.5)	72 (63.2)	16.8 (12.4)
* *p*‐value	0.67	0.51	**0.01**	**0.04**	0.07	0.1	1.00	0.52
Age								
18–52 years	20 (58.8)	15.4 (11.2)	57 (55.9)	15.2 (11.2)	34 (50.7)	13.8 (11.4)	43 (62.3)	16.6 (10.1)
> 52 years	6 (35.3)	11.4 (13.3)	61 (60.4)	15.8 (12.8)	26 (48.1)	13.7 (12.1)	41 (64.1)	16.4 (13.6)
* *p*‐value	0.11	0.11	0.52	0.92	0.77	0.79	0.83	0.5
Educational level (years of schooling)								
> 4 years	24 (52.2)	14.5 (12.0)	102 (58.6)	15.4 (11.7)	55 (51.9)	14.2 (11.6)	71 (62.3)	16.1 (11.9)
≤ 4 years	2 (40.0)	10.0 (11.5)	16 (55.2)	16.2 (13.7)	5 (33.3)	10.8 (11.8)	13 (68.4)	18.8 (13.9)
* *p*‐value	0.67	0.31	0.73	0.95	0.27	0.15	0.61	0.43
Employment status								
Employed	14 (60.9)	15.0 (12.1)	49 (62.8)	16.7 (12.3)	30 (55.6)	14.6 (10.9)	33 (70.2)	18.1 (13.5)
Unemployed	12 (42.9)	13.3 (11.9)	69 (55.2)	14.8 (11.8)	30 (44.8)	13.1 (12.2)	51 (59.3)	15.7 (11.4)
* *p*‐value	0.2	0.52	0.28	0.30	0.24	0.19	0.21	0.45
Household income (in minimum wage)								
> 1 minimum wage	21 (50.0)	14.1 (12.7)	100 (56.8)	15.2 (12.2)	53 (49.5)	13.9 (11.8)	68 (61.3)	16.1 (12.7)
≤ 1 minimum wage	5 (55.6)	13.8 (8.2)	18 (66.7)	17.4 (10.5)	7 (50.0)	12.9 (10.4)	16 (72.7)	18.8 (9.2)
* *p*‐value	1.00	0.76	0.33	0.17	0.97	0.9	0.31	0.08
Race/skin color								
White	1 (33.3)	13.0 (5.6)	8 (57.1)	19.1 (15.6)	3 (33.3)	16.3 (12.7)	6 (75.0)	20.0 (16.8)
Non‐white	25 (52.1)	14.1 (12.3)	110 (58.2)	15.2 (11.7)	57 (50.9)	13.6 (11.6)	78 (62.4)	16.3 (11.9)
* *p*‐value	0.53	0.77	0.94	0.42	0.49	0.45	0.71	0.63
Smoking habit								
Non‐smoker	17 (45.9)	13.1 (11.7)	87 (56.1)	14.7 (11.1)	41 (44.6)	12.7 (11.1)	63 (63.0)	16.1 (11.1)
Smoker/former smoker	9 (64.3)	16.5 (12.6)	31 (64.6)	18.0 (14.3)	19 (65.5)	17.4 (12.6)	21 (63.6)	17.9 (15.1)
* *p*‐value	0.24	0.40	0.30	0.29	0.05	0.07	0.95	0.96
Alcoholic beverage consumption								
No	11 (57.9)	15.4 (10.8)	49 (59.0)	16.6 (11.4)	24 (52.2)	15.0 (10.7)	36 (64.3)	17.4 (11.8)
Yes	15 (46.9)	13.3 (12.7)	69 (58.5)	14.9 (12.4)	36 (48.0)	13.0 (12.2)	48 (64.0)	16.1 (12.5)
* *p*‐value	0.45	0.30	0.94	0.17	0.65	0.16	0.97	0.38
Physical activity								
Yes	10 (45.5)	13.9 (13.4)	44 (53.0)	15.3 (11.8)	25 (46.3)	14.9 (12.9)	29 (56.9)	15.0 (11.2)
No	16 (55.2)	14.2 (11.0)	74 (61.7)	15.7 (12.1)	35 (52.2)	12.8 (10.5)	55 (67.1)	17.4 (12.8)
* *p*‐value	0.49	0.55	0.22	0.85	0.52	0.61	0.24	0.31
Hypertension								
No	22 (51.2)	14.2 (12.4)	70 (58.3)	14.8 (10.7)	43 (48.9)	13.4 (11.7)	49 (65.3)	16.1 (10.4)
Yes	4 (50.0)	13.1 (9.7)	48 (57.8)	16.5 (13.7)	17 (51.5)	14.8 (11.7)	35 (60.3)	17.0 (14.2)
* *p*‐value	1.00	0.88	0.94	0.78	0.80	0.47	0.55	0.67
Diabetes								
No	26 (51.0)	14.1 (11.9)	88 (55.0)	14.8 (10.9)	54 (48.6)	13.9 (11.4)	60 (60.0)	15.5 (10.8)
Yes	0 (0.0)	NA	30 (69.8)	18.2 (15.3)	6 (60.0)	13.2 (14.7)	24 (72.7)	19.7 (15.3)
* *p*‐value	NA	NA	0.08	0.41	0.49	0.59	0.18	0.29
Cardiovascular disease								
No	21 (48.8)	13.2 (11.3)	106 (55.8)	15.5 (12.1)	52 (47.3)	13.5 (11.5)	75 (61.0)	16.5 (12.3)
Yes	5 (62.5)	18.6 (15.0)	12 (92.3)	15.3 (9.4)	8 (72.7)	16.6 (13.5)	9 (90.0)	16.5 (9.9)
* *p*‐value	0.70	0.26	**0.01**	0.67	0.11	0.38	0.09	0.67
Last dental visit								
< 1 year	14 (48.3)	12.9 (11.5)	63 (59.4)	15.5 (12.9)	35 (50.7)	12.9 (11.7)	42 (63.6)	17.1 (13.4)
≥ 1 year	12 (54.5)	15.6 (12.6)	55 (56.7)	15.5 (10.9)	25 (48.1)	14.9 (11.6)	42 (62.7)	15.9 (10.9)
* *p*‐value	0.66	0.48	0.69	0.55	0.77	0.29	0.91	0.96
Daily toothbrushing frequency								
> 2 times	26 (51.0)	14.1 (11.9)	1 (33.3)	3.3 (3.05)	60 (49.6)	13.8 (11.6)	1 (33.3)	3.3 (3.1)
≤ 2 times	0 (0.0)	NA	117 (58.5)	15.7 (12.0)	0 (0.0)	NA	83 (63.8)	16.8 (12.2)
* *p*‐value	NA	NA	0.38	**0.03**	NA	NA	0.55	**0.02**
Daily use of dental floss								
Yes	12 (44.4)	12.4 (12.1)	42 (51.9)	14.3 (11.1)	26 (42.6)	12.9 (11.6)	28 (59.6)	15.0 (11.0)
No	14 (58.3)	15.9 (11.7)	76 (62.8)	16.2 (12.6)	34 (56.7)	14.6 (11.7)	56 (65.9)	17.3 (12.8)
* *p*‐value	0.32	0.17	0.12	0.38	0.12	0.3	0.47	0.4

*Note:* Bold numbers represent a statistically significant difference.

Abbreviations: IDF, International Diabetes Federation; NCEP, National Cholesterol Education Program; SD, standard deviation.

* *p*‐value = Statistical significance level: *p* ≤ 0.05.

In the comparison between obese and non‐obese groups according to the NCEP criterion (Table 3 and Table S1), only daily tooth brushing frequency ≤ 2 times (*p* = 0.02), presence of ≥ 2 decayed teeth (*p* = 0.04), and DMFT ≥ 16 (*p* = 0.03) were associated with OHRQoL among obese individuals.

Sequential blockwise multiple regression analysis was used to identify independent variables associated with OHRQoL according to the different obesity criteria applied (Table 4). For the analysis of the OHRQoL outcome in its dichotomous form, Poisson regression with robust variance was employed. The predictor variables that were associated with impaired OHRQoL among obese individuals and remained statistically significant, after adjusting for the others, were female sex (PR_adjusted_ = 1.51; 95% CI: 1.04–2.18—IDF criterion), cardiovascular disease (PR_adjusted_ = 1.61; 95% CI: 1.29–2.00—IDF criterion; PR_adjusted_ = 1.51; 95% CI: 1.11–2.06—NCEP criterion), and CPOD ≥ 16 (PR_adjusted_ = 1.35; 95% CI: 1.01–1.78—IDF criterion). Among non‐obese individuals, those who smoked at the time of data collection or had smoked in the past were found to be associated with greater OHRQoL impairment: PR_adjusted_ = 1.76; 95% CI: 1.15–2.71—NCEP criterion. The Hosmer‐Lemeshow goodness‐of‐fit test showed that the final results were in well‐fitting models (*p*‐values ranging from 0.87 to 1.00), indicating that the observed frequencies closely matched the expected ones.

**TABLE 4:** Sequential Blockwise Multiple Poisson Regression Analysis: Prevalence Ratios (PR) between predictor variables and oral health‐related quality of life (OHRQoL) among individuals with and without obesity, according to IDF and NCEP criteria.

Variables	Non‐obese – IDF (*n* = 51)		Obese – IDF (*n* = 203)	
	PR (95% CI)	*p* *	PR (95% CI)	*p* *
Block I (Sociodemographic variables)				
Sex: female	0.80 (0.47–1.35)	0.40	**1.51 (1.04–2.18)**	**0.03**
Age: > 52 years	0.56 (0.27–1.12)	0.10	1.10 (0.88–1.40)	0.41
Household income: ≤ 1 minimum wage	1.15 (0.65–2.05)	0.63	1.11 (0.82–1.50)	0.49
Block II (Lifestyle and oral hygiene habits)				
Alcoholic beverage consumption: yes	−0.80 (0.47–1.37)	0.42	1.06 (0.85–1.34)	0.58
Toothbrushing frequency: ≤ 2 times/day	NA	NA	1.62 (0.26–10.15)	0.52
Daily use of dental floss: no	1.35 (0.79–2.29)	0.26	1.19 (0.93–1.52)	0.18
Block III (Health and oral condition variables)				
Cardiovascular disease: yes	1.72 (0.86–3.46)	0.12	**1.61 (1.29–2.00)**	**< 0.01**
Diabetes: yes	NA	NA	1.11 (0.87–1.42)	0.32
Dental plaque index: ≥ 38%	1.05 (0.53–2.05)	0.88	1.03 (0.82–1.30)	0.86
Teeth with CAL ≥ 5 mm: ≥ 30% of teeth	0.67 (0.26–1.76)	0.43	1.18 (0.91–1.54)	0.22
Remaining teeth: ≤ 11 teeth	0.52 (0.09–2.78)	0.45	1.01 (0.75–1.33)	0.96
DMFT: ≥ 16	1.63 (0.89–3.00)	0.11	**1.35 (1.01–1.78)**	**0.04**
Decayed teeth: ≥ 2 decayed teeth	1.56 (0.90–2.71)	0.11	1.15 (0.92–1.43)	0.22

Variables	Non‐obese – NCEP (*n* = 121)		Obese – NCEP (*n* = 133)	
	PR (95% CI)	*p* *	PR (95% CI)	*p* *
Block I (Sociodemographic variables)				
Sex: female	1.40 (0.93–2.10)	0.09	0.99 (0.66–1.44)	0.91
Education level: ≤ 4 years of schooling	0.70 (0.33–1.49)	0.36	1.08 (0.76–1.51)	0.67
Household income: ≤ 1 minimum wage	0.97 (0.52–1.78)	0.91	1.18 (0.87–1.60)	0.29
Block II (Lifestyle and oral hygiene habits)				
Smoking habit: yes	**1.76 (1.15–2.71)**	**0.01**	1.01 (0.75–1.37)	0.94
Alcoholic beverage consumption: yes	0.80 (0.52–1.24)	0.33	1.01 (0.75–1.37)	0.94
Toothbrushing frequency: ≤ 2 times/day	NA	NA	2.01 (0.38–10.86)	0.41
Daily use of dental floss: no	1.40 (0.98–2.00)	0.07	1.08 (0.80–1.44)	0.6
Block III (Health and oral condition variables)				
Cardiovascular disease: yes	1.33 (0.89–2.00)	0.17	**1.51 (1.11–2.06)**	**0.01**
Diabetes: yes	1.11 (0.74–1.67)	0.6	1.16 (0.90–1.52)	0.25
Dental plaque index: ≥ 38%	0.99 (0.66–1.49)	0.97	0.85 (0.65–1.10)	0.21
Remaining teeth: ≤ 11 teeth	0.90 (0.60–1.36)	0.62	1.11 (0.83–1.48)	0.48
DMFT: ≥ 16	1.15 (0.77–1.73)	0.49	1.30 (0.96–1.74)	0.09
Decayed teeth: ≥ 2 decayed teeth	1.35 (0.95–1.98)	0.11	1.22 (0.95–1.57)	0.12

*Note:* Bold numbers represent a statistically significant difference; In each model (predictor variable under analysis + outcome = impaired OHRQoL), the remaining independent/predictor variables listed in the table were included as adjustment variables.

Abbreviations: 95% CI, 95% confidence interval; CAL, Clinical Attachment Level; DMFT, Decayed Missing and Filled Teeth; IDF, International Diabetes Federation; NA, not assessed; NCEP, National Cholesterol Education Program; PR, prevalence ratio.

* *p*‐value = statistical significance level: *p* ≤ 0.05.

When quantile regression analysis was applied, it was observed that only the predictor variable CPOD ≥ 16 (*p* = 0.02) influenced OHIP‐14 scores among obese individuals according to the IDF criterion. For the non‐obese group, the predictor variables smoking habit, non‐use of dental floss, and alcoholic beverage consumption showed a statistically significant association (*p* < 0.05) with higher OHIP‐14 scores (Table S2).

## 4 Discussion

Results of this study showed that individuals with obesity are more likely to have lower oral health‐related quality of life (OHRQoL) scores and higher OHIP‐14 scores compared to non‐obese individuals. The dimensions most affected by OHRQoL in obese individuals include physical pain (discomfort when eating), physical disability (unsatisfactory diet), psychological disability (embarrassment due to oral health condition), and social disability (irritability and difficulty performing daily activities). A previous study (Khongsirisombat et al. 2022) had also observed a negative association between impaired OHRQoL and obesity measured by the OHIP‐14. Although few studies have focused on pregnant women, previous research on pregnant women diagnosed with obesity and hypertension indicated that obese, non‐hypertensive pregnant women had higher OHIP‐14 scores compared to non‐obese, non‐hypertensive pregnant women, with some of these domains also being highlighted by the authors (Foratori‐Junior et al. 2021).

Obesity has been widely associated with poorer quality of life (Reinbacher et al. 2023; MacEwan et al. 2024), with negative impacts on both physical and psychosocial aspects. Furthermore, obesity is associated with the increased prevalence of non‐communicable chronic diseases, such as cardiovascular diseases, hypertension, diabetes, depression, and oral diseases, as well as premature mortality and economic impacts on health systems and society (MacEwan et al. 2024; Carneiro et al. 2022; Nilson et al. 2020; Kim et al. 2016).

Although systematic reviews have identified a large number of studies reporting a relationship between obesity, its treatment, and general quality of life (Kolotkin and Andersen 2017), the literature on oral health‐related quality of life in obese individuals remains limited (Khongsirisombat et al. 2022; do Prado et al. 2020). To the best of our knowledge, this study is the first to explore the predictors of OHRQoL, including clinical aspects of oral health, in adults with and without obesity. Identifying factors related to sociodemographic characteristics, lifestyle habits, and health conditions can help develop more effective public policies to promote the well‐being of obese individuals.

Among the obese individuals, the results showed that there was a significant association between female sex, cardiovascular disease, and CPOD > 16 with impaired OHRQoL, even after adjusting for covariables. The association with cardiovascular disease was consistent across multivariable models, regardless of the obesity criterion used. It is well documented that cardiovascular diseases impact OHRQoL, as oral health issues such as xerostomia, tooth loss, cavities, and periodontitis affect systemic health. Additionally, systemic inflammatory processes induced by oral bacteria can increase the risk of cardiovascular complications (Isola et al. 2023; Molania et al. 2021). The poorer OHRQoL observed among individuals with cardiovascular diseases may be related to poor oral hygiene habits. In the bivariate analysis, lower frequency of tooth brushing and higher plaque presence were associated with poorer OHRQoL, but only in the obese group. Other studies have also found higher OHIP‐14 scores in individuals with periodontitis and cardiovascular disease (Lazureanu et al. 2022), suggesting that healthcare professionals should raise awareness among these individuals about the importance of oral hygiene for improving OHRQoL.

Poor oral health, as evidenced by a high CPOD index, suggests that the greater the severity of dental caries, the worse is the quality of life in obese individuals. Dental caries and missing teeth directly impact masticatory function, cause physical discomfort and lower self‐esteem. Studies have shown a significant correlation between the presence of dental caries and oral health‐related quality of life (Molania et al. 2021; Khalifa et al. 2020). Masticatory difficulties can lead to the restriction of nutritious foods, such as vegetables, or to overcooking foods, which reduces their nutritional value and may contribute to malnutrition or obesity (Moynihan and Kelly 2014). Tooth loss and other oral limitations can impair masticatory ability, leading to dietary adaptations, with a preference for softer foods, which can contribute to weight gain and exacerbate obesity (Wu et al. 2025; Passos‐Soares et al. 2018).

Furthermore, studies indicate that individuals with high CPOD scores or few remaining teeth are at greater risk of being overweight and obese, especially when they do not use adequate dental prostheses (Huang et al. 2025). Diets low in fiber and micronutrients, resulting from food restrictions imposed by oral health problems, negatively affect overall health and well‐being (Moynihan and Kelly 2014). These findings reinforce the idea that oral health should be viewed not only from a functional and aesthetic perspective but also as a crucial factor for healthy eating, nutritional status, and oral health‐related quality of life.

Regarding the association between OHRQoL and female sex, other studies support these findings (Koistinen et al. 2020; Tsakos et al. 2009). Notably, the association with sex was observed in both the obese and non‐obese groups. Previous studies have suggested that impaired quality of life in women may be related to higher proportions of dental anxiety in this group (Galletti et al. 2024).

In non‐obese individuals, in addition to female sex, smoking, alcohol consumption, and lack of dental floss use stood out as factors associated with worse OHRQoL. Individuals with these harmful habits and inadequate oral hygiene practices tend to report worse oral health‐related quality of life (Tenani et al. 2021; Colaço et al. 2020). The presence of comorbidities associated with obesity and the consequent use of medications to manage these chronic conditions can influence lifestyle changes, which may explain the absence of association between these habits and OHRQoL in obese individuals.

Although the literature suggests an association between obesity and periodontitis, with both conditions sharing a chronic inflammatory state, the present study did not find a significant association between periodontitis and obesity, nor between periodontitis and OHRQoL. This may be explained by the relatively small sample size with respect to the obese group and by high tooth loss, which may have led to the underestimation of periodontitis.

This study is limited by its cross‐sectional design, which precludes the identification of a temporal relationship between exposure and outcome. Additionally, while the sample size (*N* = 250) was sufficient for the planned exploratory analyses, it required a parsimonious approach to the multivariate models to avoid overfitting. Regarding the obesity assessment, we acknowledge that while waist circumference (WC) and height were recorded, the Waist‐to‐Height Ratio (WtHR) was not included in the present analysis. The decision to focus exclusively on WC was based on the original research protocol and the aim to ensure comparability with existing literature that predominantly uses WC as a proxy for visceral adiposity. Future studies with larger cohorts should consider WtHR as a potentially more sensitive metric for central obesity in this context.

Furthermore, the use of two distinct obesity criteria (IDF and NCEP‐ATPIII) highlighted that the associations between obesity and OHRQoL may vary depending on the severity and clinical definition of abdominal adiposity used (Capuno et al. 2022). This dual approach, while adding complexity, reinforces the reliability of our findings by demonstrating consistent impacts across different validated thresholds of central obesity.

One of the main strengths of the study was the use of a sequential blockwise model of analysis, which respects the distal‐proximal logic between social determinants and health outcomes. This approach allows for a better understanding of how sociodemographic factors indirectly influence OHRQoL by mediating health practices, lifestyle, and clinical oral health conditions. By considering different levels of influence, the study avoids the error of overestimating direct associations and properly controls for confounding factors. This methodological choice contributes to the greater reliability of the findings and provides a more accurate interpretation of the associations found.

The results suggest that oral health assessments should be integrated into obesity management. It is recommended that Primary Health Care services adopt protocols that include OHRQoL screening (e.g., using the OHIP‐14) during routine examinations. Furthermore, training professionals to identify early oral changes, along with providing nutritional guidance, may facilitate referrals for specialized dental evaluations. This integrated approach, promoting both oral health and weight control, has the potential to significantly improve the quality of life of obese individuals and optimize resources in public health services.

In conclusion, obese individuals exhibit a greater impairment of OHRQoL compared to non‐obese individuals. This impairment is influenced by factors such as female gender, the presence of cardiovascular disease, and an elevated CPOD. The domains of physical pain, physical incapacity, psychological incapacity, and social incapacity are the most affected in individuals with obesity. Analyzing the subjective measurement of OHRQoL in relation to socioeconomic factors, lifestyle habits, health conditions, and clinical oral aspects provides a greater understanding of the relationships between nutritional status and the psychosocial status of the participants, highlighting the complexity of the challenges faced by this population.

## Author Contributions

All authors have made substantial contributions to the conception and design of the study. A.K.O.S.B., I.S.G.‐F., S.S.C., S.C.T., S.S.M., A.C.M.G.F., D.O.C., M.C.A.M.S., I.S.S., E.M.M.C., J.E.T.B., A.M.H., and J.S.P.‐S. have been involved in data collection and data analysis. A.K.O.S.B., I.S.G.‐F., S.S.C., S.C.T., S.S.M., A.C.M.G.F., E.M.M.C., J.E.T.B., A.M.H., G.J.S., F.A.S., P.M.L., and J.S.P.‐S. have been involved in data interpretation, drafting the manuscript, and revising it critically and have given final approval for the version to be published.

## Conflicts of Interest

The authors declare no conflicts of interest.

## Supporting information


**Table S1:** Frequency distribution and mean oral health‐related quality of life (OHRQoL), according to clinical oral variables among individuals with and without obesity, based on IDF and NCEP criteria.
**Table S2:** Quantile Regression Coefficients (50th Percentile) for the Association between Predictor Variables and OHIP‐14 Scores, Stratified by Obesity Status (IDF and NCEP Criteria).

## Data Availability

The data that support the findings of this study are available on request from the corresponding author. The data are not publicly available due to privacy or ethical restrictions.

## Endnotes

^1^
Statistical Package for the Social Sciences—SPSS for Windows, version 22.0, and STATA Statistical Software—version 18.0.
