# Supplementary material for: Obesity and Oral Health‐Related Quality of Life: An Exploratory Analysis of Associated Factors
Source: Oral Dis. 2026 Apr 10;32(9):2932–45. doi: 10.1111/odi.70326 (PMC13752622; doi:10.1111/odi.70326)
Supplement: Supplementary file 1 [file ODI-32-2932-s001.docx]

**Supplementary Material**

**Table 1.** Frequency distribution and mean oral health-related quality of life (OHRQoL), according to clinical oral variables among individuals with and without obesity, based on IDF and NCEP criteria.

|  | **Non-obese – IDF**  **(n = 51)** | | **Obese – IDF**  **(n = 203)** | |
| --- | --- | --- | --- | --- |
| **Variables** | **Compromised OHRQoL** | **Mean (±SD)** | **Compromised OHRQoL** | **Mean (±SD)** |
| **Teeth with CAL 3–4 mm** | | | | |
| <30% of teeth | 11 (50.0) | 15.4 (15.1) | 35 (56.5) | 16.4 (12.5) |
| ≥30% of teeth | 15 (51.7) | 13.1 (9.1) | 83 (58.9) | 15.1 (11.8) |
| p-value | 0.90 | 0.93 | 0.75 | 0.45 |
| **Teeth with CAL ≥5 mm** | | | | |
| <30% of teeth | 21 (55.3) | 15.2 (12.6) | 80 (55.2) | 14.1 (10.1) |
| ≥30% of teeth | 5 (38.5) | 10.9 (9.4) | 38 (65.5) | 19.0 (15.4) |
| p-value | 0.30 | 0.25 | 0.18 | 0.13 |
| **Teeth with probing depth ≥4 mm** | | | | |
| <4 teeth | 20 (55.6) | 13.9 (12.0) | 80 (59.3) | 15.5 (11.9) |
| ≥4 teeth | 6 (40.0) | 16.6 (12.7) | 38 (55.9) | 16.7 (20.8) |
| p-value | 0.31 | 0.68 | 0.65 | 0.86 |
| **Bleeding on probing** | | | | |
| <10% | 15 (55.6) | 14.8 (12.4) | 48 (55.2) | 15.3 (11.9) |
| ≥10% | 11 (45.8) | 13.1 (11.6) | 70 (60.3) | 15.6 (12.1) |
| p-value | 0.48 | 0.69 | 0.46 | 0.9 |
| **Dental plaque index** | | | | |
| <38% | 14 (58.3) | 14.8 (10.5) | 55 (55.6) | 13.2 (10.5) |
| ≥38% | 12 (44.4) | 13.4 (13.3) | 63 (60.6) | 17.7 (12.9) |
| p-value | 0.32 | 0.4 | 0.46 | **0.01** |
| **Remaining teeth** | | | | |
| >11 teeth | 25 (53.2) | 14.6 (11.9) | 92 (55.1) | 14.1 (10.6) |
| ≤11 teeth | 1 (25.0) | 7.8 (11.0) | 26 (72.2) | 21.8 (20.5) |
| p-value | 0.28 | 0.15 | **0.05** | **0.01** |
| **Presence of caries** | | | | |
| <2 decayed teeth | 14 (43.8) | 12.9 (9.6) | 65 (53.3) | 13.6 (11.4) |
| ≥2 decayed teeth | 12 (63.2) | 15.9 (15.3) | 53 (65.4) | 18.4 (12.4) |
| p-value | 0.18 | 0.65 | 0.09 | 0.08 |
| **Caries severity** | | | | |
| DMFT <16 | 14 (50.0) | 13.2 (10.4) | 41 (47.1) | 13.4 (10.9) |
| DMFT ≥16 | 12 (52.2) | 15.1 (13.8) | 77 (66.4) | 16.9 (12.6) |
| p-value | 0.88 | 0.92 | **0.01** | **0.05** |
| **Periodontitis** | | | | |
| No | 9 (52.9) | 14.3 (13.1) | 29 (61.7) | 15.0 (8.9) |
| Yes | 17 (50.0) | 13.9(11.6) | 89 (57.1) | 16.0 (12.8) |
| p-value | 0.84 | 0.88 | 0.57 | 0.63 |
|  | **Non-obese - NCEP**  **(n=121)** | | **Obese - NCEP**  **(n=133)** | |
| **Variables** | **Compromised OHRQoL** | **Mean (±SD)** | **Compromised OHRQoL** | **Mean (±SD)** |
| **Teeth with CAL 3–4 mm** | | | | |
| <30% of teeth | 20 (42.6) | 14.5 (15.6) | 26 (70.3) | 18.2 (12.5) |
| ≥30% of teeth | 40 (54.1) | 13.4 (10.3) | 58 (60.4) | 15.8 (12.1) |
| p-value | 0.22 | 0.94 | 0.29 | 0.23 |
| **Teeth with CAL ≥5 mm** | | | | |
| <30% of teeth | 44 (48.9) | 13.1 (11.2) | 57 (61.3) | 15.5 (9.8) |
| ≥30% of teeth | 16 (51.6) | 15.7 (12.7) | 27 (67.5) | 18.9 (16.3) |
| p-value | 0.79 | 0.40 | 0.50 | 0.80 |
| **Teeth with probing depth ≥4 mm** | | | | |
| <4 teeth | 42 (50.6) | 13.7(11.7) | 58 (65.9) | 16.5 (12.1) |
| ≥4 teeth | 18 (47.4) | 16.7 (12.7) | 26 (57.8) | 16.7 (20.8) |
| p-value | 0.74 | 0.72 | 0.36 | 0.78 |
| **Bleeding on probing** | | | | |
| <10% | 26 (44.8) | 13.2 (12.1) | 37 (66.1) | 17.3 (11.6) |
| ≥10% | 34 (54.0) | 14.4 (11.3) | 47 (61.0) | 15.9 (12.6) |
| p-value | 0.32 | 0.33 | 0.55 | 0.29 |
| **Dental plaque index** | | | | |
| <38% | 29 (46.8) | 12.6 (10.9) | 40 (65.6) | 14.3 (10.0) |
| ≥38% | 31 (52.5) | 14.9 (12.3) | 44 (61.1) | 18.4 (13.5) |
| p-value | 0.52 | 0.4 | 0.59 | 0.13 |
| **Remaining teeth** | | | | |
| >11 teeth | 51 (48.1) | 13.2 (11.2) | 66 (61.1) | 15.3 (10.5) |
| ≤11 teeth | 9 (60.0) | 18.3 (14.0) | 18 (72.0) | 21.7 (16.9) |
| p-value | 0.39 | 0.21 | 0.31 | 0.14 |
| **Presence of caries** | | | | |
| <2 decayed teeth | 34 (45.9) | 11.9 (9.9) | 45 (56.3) | 14.8(11.8) |
| ≥2 decayed teeth | 26 (55.3) | 16.7(13.5) | 39 (73.6) | 19.0(12.5) |
| p-value | 0.32 | 0.08 | **0.04** | **0.03** |
| **Caries severity** | | | | |
| DMFT <16 | 23 (41.8) | 11.8 (9.9) | 32 (53.3) | 15.1 (11.3) |
| DMFT ≥16 | 37 (56.1) | 15.5 (12.7) | 52 (71.2) | 17.7 (12.9) |
| p-value | 0.12 | 0.16 | **0.03** | 0.21 |
| **Periodontitis** | | | | |
| No | 18 (52.9) | 13.7 (11.6) | 20 (66.7) | 16.1(7.9) |
| Yes | 42 (48.3) | 13.8 (11.7) | 64 (62.1) | 16.6 (13.2) |
| p-value | 0.65 | 0.9 | 0.65 | 0.51 |

DP = standard deviation; * P-value = Statistical significance level: p ≤ 0.05; Bold numbers represent a statistically significant difference; International Diabetes Federation (IDF); National Cholesterol Education Program (NCEP); CAL = Clinical Attachment Level; DMFT = Decayed, Missing, and Filled Teeth.

**Table 2.** Quantile Regression Coefficients (50th Percentile) for the Association between Predictor Variables and OHIP-14 Scores, Stratified by Obesity Status (IDF and NCEP Criteria).

|  | **Non-obese**  **IDF (n=51)** | | **Obese**  **IDF (n=203)** | |
| --- | --- | --- | --- | --- |
|  | **Regression coefficient**  **(95% CI)** | **P*** | **Regression coefficient**  **(95% CI)** | **P*** |
| Sex: female | 0.25 (-10.06; 10.57) | 0.96 | 3.17 (-2.99; 9.32) | 0.31 |
| Age: >52 years | -7.62 (-18.15; 2.91) | 0.15 | -0.83 (6.50; 4.84) | 0.77 |
| Household income: ≤ 1 minimum wage | -1.25 (-13.92; 11.42) | 0.84 | 2.5 (-4.72; 9.72) | 0.5 |
| Alcoholic beverage consumption: yes | -9.87 (-20.08;0.33) | 0.06 | -2.33 (-7.48; 2.81) | 0.37 |
| Toothbrushing frequency: ≤2 times/day | NA | NA | 6.33 (-13.96; 26.62) | 0.54 |
| Daily use of dental floss: no | 6.25 (-2.49; 14.98) | 0.16 | 0.66 (-4.36;5.69) | 0.79 |
| Diabetes: yes | NA | NA | -1.00 (-7.19; 5.19) | 0.75 |
| Teeth with CAL ≥5 mm: ≥ 30% of teeth | 0.12 (-13.08; 13.33) | 0.99 | 4.33 (-1.99; 10.66) | 0.18 |
| Remaining teeth: ≤11 teeth | -5.63 (-25.86; 14.61) | 0.58 | 6.00 (-1.37; 13.37) | 0.11 |
| DMFT ≥16 | 6.13 (-4.95;17.19) | 0.27 | 1.67 (-3.85; 7.18) | 0.55 |
| Presence of decayed teeth: ≥ 2 decayed teeth | 2.38 (-7.36;12.11) | 0.63 | 6.00 (1.02; 10.97) | **0.02** |
|  | **Non-obese**  **NCEP (n=121)** | | **Obese**  **NCEP (n=133)** | |
|  | **Regression coefficient**  **(95% CI)** | **P*** | **Regression coefficient**  **(95% CI)** | **P*** |
| Sex: female | 6.00 (-0.48; 12.48) | 0.07 | -3.81 (13.42; 5.79) | 0.43 |
| Education level: ≤4 years of schooling | -3.00 (-12.00; 6.06) | 0.51 | -1.90 (-11.75; 7.93) | 0.70 |
| Household income: ≤ 1 minimum wage | -1.00 (-10.21; 8.21) | 0.83 | 7.82 (-1.00; 16.64) | 0.08 |
| Smoking habit: yes | 8.00 (0.61; 15.39) | **0.03** | 1.27 (-6.74; 9.29) | 0.75 |
| Alcoholic beverage consumption: yes | -7.00 (-13.58; -0.42) | **0.04** | -1.91 (-8.93; 5.11) | 0.59 |
| Toothbrushing frequency: ≤2 times/day | NA | NA | 8.27 (-14.61; 31.16) | 0.48 |
| Daily use of dental floss: no | 7.00 (1.18; 12.82) | **0.02** | -0.01 (-7.06; 6.88) | 0.98 |
| Diabetes: yes | -6.00 (-16.48; 4.48) | 0.26 | 1.27 (-6.70; 9.24) | 0.75 |
| Remaining teeth: ≤11 teeth | 1.00 (-8.67; 10.67) | 0.84 | 5.63 (-3.89; 15.16) | 0.24 |
| DMFT ≥16 | 1.00 (-5.23; 7.24) | 0.75 | 0.18 (-7.04; 7.40) | 0.96 |
| Presence of decayed teeth: ≥ 2 decayed teeth | 6.00 (-0.23;12.24) | 0.06 | 4.36 (-2.40; 11.13) | 0.20 |

95% CI = 95% confidence interval; NA = not assessed; * P-value = statistical significance level: p ≤ 0.05; Bold numbers represent a statistically significant difference; IDF = International Diabetes Federation; NCEP = National Cholesterol Education Program; CAL = Clinical Attachment Level; DMFT = Decayed, Missing, and Filled Teeth; In each model (predictor variable under analysis + outcome = impaired OHRQoL), the remaining independent/predictor variables listed in the table were included as adjustment variables.
